# An Ultra-Performance Liquid Chromatography–Tandem Mass Spectrometric Method for the Simultaneous Determination of Eighteen Marker Compounds in the Traditional Herbal Formula Bopyeo-Tang

**DOI:** 10.3390/ph17030352

**Published:** 2024-03-08

**Authors:** Chang-Seob Seo

**Affiliations:** KM Science Research Division, Korea Institute of Oriental Medicine, Daejeon 34054, Republic of Korea; csseo0914@kiom.re.kr; Tel.: +82-42-868-9361

**Keywords:** simultaneous analysis, traditional herbal formula, Bopyeo-tang, UPLC–MS/MS

## Abstract

Bopyeo-tang (BPT), comprising six medicinal plants, has been used for the treatment of respiratory diseases such as pulmonary fibrosis and chronic obstructive pulmonary disease. In this study, we developed and validated a quantitative method for the quality assessment of BPT using ultra-performance liquid chromatography with tandem mass spectrometry (UPLC–MS/MS). Eighteen marker compounds were separated on an Acquity UPLC BEH C_18_ reversed-phase column (2.1 mm × 100 mm, 1.7 μm) via gradient elution with a 0.1% aqueous formic acid–acetonitrile mobile phase. The multiple-reaction monitoring mode was used to improve analysis speed and accuracy. The coefficients of determination, limits of detection, and limits of quantitation of the 18 marker compounds were 0.9991–0.9996, 0.36–24.45 μg/L, and 1.07–73.35 μg/L, respectively. The recovery was 85.19–110.25%, and the relative standard deviation of precision was ≤9.01%. When applied to a typical BPT sample, the method revealed a range of concentrations from below the quantitative limit (one compound only) to a maximum of 3.20 mg/freeze-dried g. This method will be used for quality control of BPT preparations.

## 1. Introduction

Bopyeo-tang (BPT), called Bufei-tang or simply Bufei in China, is a traditional herbal formula comprising parts or extracts of six medicinal plants: *Morus alba* L. (Mori Radicis Cortex), *Rehmannia glutinosa* (Gaertn.) DC. (Rehmanniae Radix Preparata), *Panax ginseng* C.A.Mey. (Ginseng Radix), *Aster tataricus* L.f. (Asteris Radix et Rhizoma), *Astragalus propinquus* Schischkin (Astragali Radix), and *Schisandra chinensis* (Turcz.) Baill. (Schisandrae Fructus). BPT has been widely used in the clinical treatment of respiratory diseases such as chronic obstructive pulmonary disease (COPD) [1,2,3].

COPD is a disease that causes airflow obstruction and breathing problems, with symptoms such as chronic coughing, phlegm production, wheezing, and dyspnea [2,4]. The disease kills approximately three million people worldwide a year. Smoking and exposure to harmful substances are the main causes; both are irreversible risk factors from exposure [2,4,5]. The worldwide prevalence of COPD is estimated at between 7.5% and 10% [6].

Among the many herbal formulas used as medicines, Yupingfeng san, the Bufei Yishen formula, the Jia-Wei-Bu-Shein-Yi-Qi formula, and San-Huang Gu-Ben Zhi-Ke have each been used to treat COPD [7,8,9,10]. All these formulas contain Astragali Radix. Astragali Radix extract, Astragali Radix polysaccharide fraction, and astragaloside, a major component of Astragali Radix, were reported to be effective when used as COPD treatments [11,12,13]. Mori Radicis Cortex, Rehmanniae Radix Preparata, Ginseng Radix, Asteris Radix et Rhizoma, and Schisandrae Fructus—which are other components of BPT—have also been reported to be effective as COPD treatments [14,15]. In addition to its efficacy against COPD, positive effects on lung cancer and pulmonary fibrosis have also been demonstrated [16,17,18,19].

We conducted a study to establish and verify an analytical method for the quality control of traditional herbal formulas using high-performance liquid chromatography or ultra-performance liquid chromatography with tandem mass spectrometry (UPLC–MS/MS) [20,21,22,23,24]. However, the only analytical study on BPT was conducted by He et al. [18]. This is the only study using a high-performance liquid chromatography–diode array detection electrospray ionization–hybrid ion trap–time of flight mass spectrometry method, detecting 89 peaks from BPT and identifying 47 of these [18]. In addition, they identified acetylene glycosides (from Cononopsis Radix), flavonoids and saponins (Astragali Radix), lignan derivatives (Schisandrae Fructus), organic acidic and iridoid compounds (Rehmanniae Radix Preparata), organic acidic and peptides compounds (Asteris Radix et Rhizoma), and styrenes (Mori Radicis Cortex). However, they used BPT containing Cononopsis Radix instead of Ginseng Radix, but more importantly, each analysis takes approximately 150 min [18]. Therefore, there is scope for a BPT analysis method capable of quantitatively analyzing BPT’s major components in a shorter time.

In the present study, a quantitative and simultaneous analyzing method for the efficient quality control of BPT is described and validated using a more sensitive and accurate technique—ultra-performance liquid chromatography with tandem mass spectrometry (UPLC–MS/MS). The marker analytes for simultaneous analysis were mulberroside A, hydroxymethylfurfural, chlorogenic acid, rutin, calycosin 7-O-glucoside, isoquercetin, 3,4-dicaffeoylquinic acid, 3,5-dicaffeoylquinic acid, ginsenoside Rg_1_, resveratrol, calycosin, quercetin, kaempferol, ginsenoside Rb_1_, astragaloside IV, schizandrin, gomisin A, and gomisin N.

## 2. Results and Discussion

### 2.1. Selection of Marker Compounds for Quality Assessment of BPT Using the UPLC–MS/MS Multiple-Reaction Monitoring (MRM) Method

Most traditional herbal formulas are complex preparations consisting of two or more different herbal medicines, contain numerous ingredients, and show various targets and various effects. Therefore, a scientific approach is needed to evaluate the quality of these traditional herbal formulas.

In this study, to develop a simultaneous determination analysis method using UPLC–MS/MS, the 18 dominant compounds comprising BPT were chosen as markers. These were mulberroside A, chlorogenic acid, rutin, isoquercetin, and resveratrol from *M. alba* [25,26,27], hydroxymethylfurfural from *R. glutinosa* [28], ginsenoside Rb_1_ and ginsenoside Rg_1_ from *P. ginseng* [29], chlorogenic acid, isochlorogenic acid A, isochlorogenic acid B, quercetin, and kaempferol from *A. tataricus* [30,31], astragaloside IV, calycosin, and calycosin 7-O-glucoside from *A. propinquus* [32,33], and schizandrin, gomisin A, and gomisin N from *S. chinensis* [34].

### 2.2. Identification of the 18 Marker Compounds via the UPLC–MS/MS MRM Method

**Figure 1:**
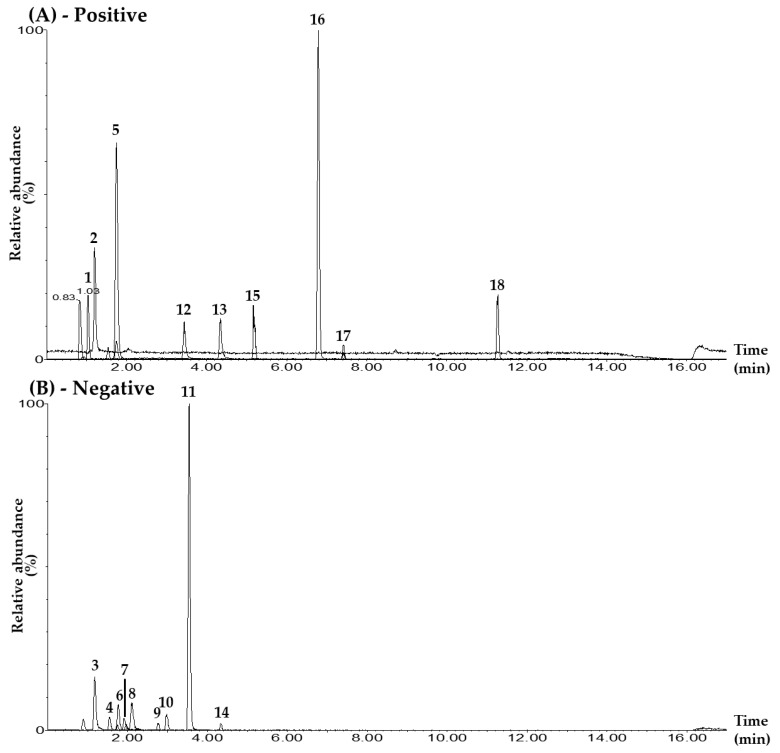

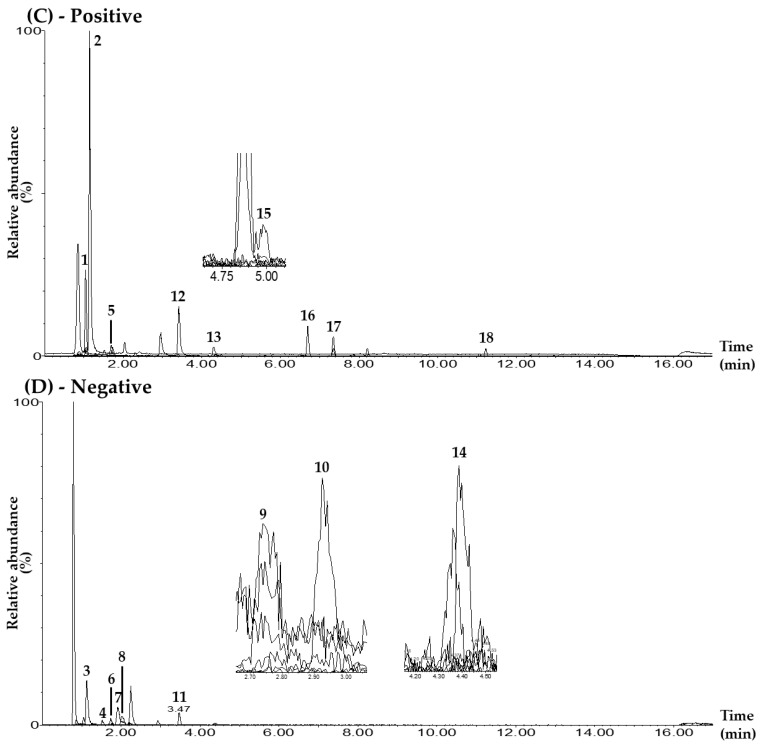
Representative total ion chromatograms of the mixed standard solution (**A**,**B**) and the BPT sample (**C**,**D**) under the UPLC–MS/MS MRM method in positive-ion (**A**,**C**) and negative-ion (**B**,**D**) modes. Mulberroside A (1), hydroxymethylfurfural (2), chlorogenic acid (3), rutin (4), calycosin 7-O-glucoside (5), isoquercetin (6), isochlorogenic acid B (7), isochlorogenic acid A (8), ginsenoside Rg_1_ (9), resveratrol (10), calycosin (11), quercetin (12), kaempferol (13), ginsenoside Rb_1_ (14), astragaloside IV (15), schizandrin (16), gomisin A (17), and gomisin N (18). The concentrations of each compound in the mixed standard solution were as follows: 10.00 μg/L (hydroxymethylfurfural, chlorogenic acid, rutin, calycosin 7-O-glucoside, isoquercetin, isochlorogenic acid A, resveratrol, calycosin, kaempferol, schizandrin, gomisin A, and gomisin N); 50.00 μg/L (quercetin); 100.00 μg/L (isochlorogenic acid B); 500.00 μg/L (ginsenoside Rb_1_); 2500.00 μg/L (mulberroside A); and 5000.00 μg/L (ginsenoside Rg_1_ and astragaloside IV).

In the UPLC–MS/MS electrospray ionization mode, 9 of the 18 markers—mulberroside A, hydroxymethylfurfural, calycosin 7-O-glucoside, quercetin, kaempferol, astragaloside IV, schizandrin, gomisin A, and gomisin N—were detected in positive-ion mode as [M+H]^+^. The other nine compounds—chlorogenic acid, rutin, iosquercetin, isochlorogenic acid B, isochlorogenic acid A, ginsenoside Rg_1_, resveratrol, calycosin, and ginsenoside Rb_1_—were detected in negative-ion mode as the [M–H]^−^ form (Table 1, Figure 1, Figures S1 and S2).

For quantitative analysis, the conditions of the MRM transitions (precursor ion (Q1) and product ion (Q3)) of each marker compound were set as shown in Table 1 and Figure S3. The MRM analysis method has the advantage of being able to quantify multiple compounds simultaneously by setting the cone voltage and collision energy for each compound separately in UPLC–MS/MS analysis. The Q3 peaks of mulberroside A and isoquercetin were set to *m*/*z* 245.0 and 300.0 in the form of [M+H–2Glu]^+^ and [M–H–Glu]^–^, respectively, where two glucose groups and one glucose group were lost from each Q1 peak [35,36]. The Q3 peaks of hydroxymethylfurfural, schizandrin, and gomisin A were set to the [M+H–H_2_O]^+^ form generated at *m*/*z* 109.0, 415.0, and 399.0 via the loss of one water molecule from each Q1 peak, respectively [37,38]. The Q3 peak for chlorogenic acid was set at *m*/*z* 191.0 for the [M–H–C_9_H_7_O_3_]^–^ form, with the caffeoyl group removed from the Q1 peak [39]. The Q3 peaks for isochlorogenic acid B and astragaloside IV were set at *m*/*z* 173.0 and 142.9 for the [M–H–2caffeoyl]^–^ and [M+H–C_33_H_53_O_12_]^+^ forms, where two caffeoyl groups and the glucose-xylose-C_22_H_33_O_2_ functional group were removed from the Q1 peak at *m*/*z* 515.5 and 785.4, respectively [39,40]. For the three compounds, rutin, isochlorogenic acid A, and gomisin N, Q3 peaks were set to [M–H–Rhm–Glc]^–^, [Caffeoyl group]^–^, and [M+H–(OCH_3_–H)]^+^ forms at *m*/*z* 300.5, 352.7, and 371.0, respectively [36,39,41]. The Q3 peaks of calycosin 7-O-glucoside and ginsenoside Rg_1_ were set to *m*/*z* 285.1 and 636.8, respectively, in the form of [M+H–Glu]^+^ and [M–H–Glu]^–^, in which a glucose molecule was lost from each Q1 peak [42,43]. The Q3 peaks for resveratrol and calycosin were set at *m*/*z* 185.0 and 267.9 for the [M–H–C_2_H_2_O]^–^ and [M–H–CH_3_]^−^ forms, where the C_2_H_2_O moiety and methyl group were removed from the Q1 peak at *m*/*z* 227.2 and 283.4, respectively [44,45]. For MRM transitions of the flavonoids quercetin and kaempferol, the Q3 peaks were detected at *m*/*z* 153.0 and 153.2, and were both generated via the cleavage of the C-ring through the retro Diels–Alder reaction [46]. For the MRM transition of ginsenoside Rb_1_, the peak at *m*/*z* 179.0—where glucose was detected in the Q1 peak—was selected as the Q3 peak [47].

### 2.3. Validation of the UPLC–MS/MS MRM Method

In the developed analytical method, validation is a very important factor in the standardization of traditional herbal formulas. This is necessary to ensure the ultimate validity, practicality, and reproducibility of the scientific methods used in the developed assay.

The regression equations and values of the coefficient of determination (*r*^2^), limit of detection (LOD), and limit of quantitation (LOQ) tested for different concentrations of each marker are summarized in Table 2. The *r*^2^ values spanned from 0.9991 to 0.9996, showing high linearity, and the LOD and LOQ concentrations were 0.36–24.45 μg/L and 1.07–73.35 μg/L, respectively. Table 3 shows the recovery, precision, and repeatability results for the markers. The recoveries were 85.19–110.25% (relative standard deviation (RSD) ≤ 6.60%), which were within the acceptable range of 80–120%. The RSD value of precision was ≤9.01%, which was within the tolerance level of ±20%. In the stability test, these 18 marker compounds were considered stable for at least 3 days as the change in peak area content was less than 3.0% within 3 days (Table 3).

### 2.4. Simultaneous Determination of the 18 Marker Compounds in a BPT Sample via the UPLC–MS/MS MRM Method

The new method was applied for the simultaneous determination of the 18 compounds in a BPT sample (Table 4). Among these compounds, gomisin A was detected below the LOQ. The other compounds were detected at 0.001–3.20 mg/freeze-dried g of BPT. Hydroxymethylfurfural—a major component of *R. glutinosa*—was by far the most abundant compound, at 3.20 mg/g, in this BPT sample.

## 3. Materials and Methods

### 3.1. Plant Materials

The six raw medicinal herbs that BPT is composed of—Mori Radicis Cortex (Moraceae, China), Rehmanniae Radix Preparata (Plantaginaceae, Gunwi, Republic of Korea), Ginseng Radix (Araliaceae, Geumsan, Republic of Korea), Asteris Radix et Rhizoma (Compositae, China), Astragali Radix (Lequminosae, Jecheon, Republic of Korea), and Schisandrae Fructus (Schisandraceae, Samcheok, Republic of Korea)—were purchased from Kwangmyungdang Pharmaceutical (Ulsan, Republic of Korea) in 2018. They were used for this research after morphological identity confirmation by Dr. Goya Choi, a herbalist at the Korea Institute of Oriental Medicine (KIOM, Daejeon, Republic of Korea). Six crude material specimens (CA05–1 to CA05–6) were kept at the KM Science Research Division, KIOM.

### 3.2. Chemicals and Reagents

The 18 reference marker compounds (Figure S4) used for simultaneous determination in BPT via the UPLC–MS/MS system were purchased from high-purity natural product manufacturers such as EnsolBioSciences (Daejeon, Republic of Korea), Merck KGaA (Darmstadt, Germany), Shanghai Sunny Biotech (Shanghai, China), Biopurify Phytochemicals (Chengdu, China), and Wuhan ChemFaces Biochemical (Wuhan, China). Detailed information on these marker compounds is summarized in Table S1. LC–MS-grade organic solvents (methanol and acetonitrile) and reagents (formic acid) were purchased from Thermo Fisher Scientific (Cleveland, OH, USA). Ultrapure deionized water was obtained using the Elix Technology Inside system (Milli-Q Integral 15, Merck, Millipore, France).

### 3.3. Preparation of a BPT Water Extract

A BPT water extract was prepared by KIOM based on previously reported extraction protocols [20,21,22,23,24]. Briefly, Mori Radicis Cortex (1500 g, Moraceae, China), Rehmanniae Radix Preparata (1500 g, Scrophulariaceae, Gunwi, Korea), Ginseng Radix (500 g, Ariliaceae, Geumsan, Republic of Korea), Asteris Radix et Rhizoma (500 g, Compositae, China), Astragali Radix (500 g, Leguminosae, Jecheon, Republic of Korea), and Schisandrae Fructus (500 g, Schisandraceae, Samcheok, Republic of Korea) were well mixed and extracted with 50 L of (deionized) water in a COSMOS-660 extractor (Kyungseo E&P, Incheon, Republic of Korea) at 100 °C for 2 h. The cooled extract was lyophilized using an LP100R freeze-dryer (IlShinBioBase, Dongducheon, Republic of Korea) to obtain a powder sample (1600 g, a 32% yield) that was held at −20 °C before use.

### 3.4. Analytical Conditions for the Simultaneous Quantification of Markers in a BPT Sample via the UPLC–MS/MS MRM Method

The simultaneous quantification of the 18 marker compounds was performed by modifying analytical protocols previously reported to the UPLC–MS/MS system comprising a Waters Acquity UPLC I-Class Plus system and a Xevo TQ-XS triple quadrupole mass spectrometry system (Waters, Milford, MA, USA) [23,24]. The chromatographic separation of all markers was carried out using a 0.1% (*v*/*v*) aqueous formic acid–acetonitrile mobile phase system and an Acquity UPLC BEH reverse-phase column (Waters) maintained at 45 °C. Detailed operating conditions for the UPLC and mass spectrometry systems are described in Table 5. In addition, the operating parameters for the MRM of each compound are listed in Table 1.

Standard stock solutions of each reference standard compound were prepared at a concentration of 1.0 mg/mL using methanol, stored in a refrigerator (approximately 4 °C), and used for analysis.

The sample solution for UPLC–MS/MS analysis was prepared at a concentration of 0.05 mg/mL with 70% methanol. That is, we took exactly 0.5 mg of BPT sample, placed it in a 10 mL volumetric flask, and added 70% methanol. The mixed sample solution was continuously subjected to ultrasonic extraction for 5 min and vortexing for 1 min. The solution was filtered through a 0.22 μm polytetrafluoroethylene hydrophobic membrane filter (catalog No. SSKPTFE13022B, SsolKorea, Daejeon, Republic of Korea) before use in UPLC–MS/MS analysis.

### 3.5. Validation of the UPLC–MS/MS MRM Method

For validation linearity, sensitivities (LOD and LOQ), accuracy, and precision parameters were evaluated using the International Conference on Harmonisation guidelines, specifically the methods for there Q2B validation of analytical procedures [48]. Briefly, linearity was evaluated using the *r*^2^ value of the calibration regression equation for each marker compound. The sensitivities, LOD and LOQ, were calculated from signal-to-noise ratios of 3:1 and 10:1, respectively.

Recovery was determined using a standard addition method. In general, it is difficult to source reference samples to be used as blanks in herbal medicine analysis that do not contain marker components. Therefore, in this study, recoveries were calculated by adding the authentic compounds the BPT extract at three concentrations (low, medium, and high) and subtracting the endogenous quantity in the sample. The calculation equation was as follows:

$$Recovery\left(\%\right)=\frac{found\;amount-original\;amount}{spiked\;amount}\times 100$$

Intraday and interday precisions were determined in the same way recovery was, within one day and over three consecutive days, and then evaluated as RSD (%), which was calculated as follows:

$$RSD\left(\%\right)=\frac{standard\;deviation}{mean}\times 100$$

Finally, the stability of 18 marker compounds was tested for 3 days at room temperature (23 ± 1 °C) using the sample solution and was evaluated using the RSD values.

## 4. Conclusions

We developed and validated a fast, sensitive, and accurate UPLC–MS/MS MRM method to quantify the quality of BPT, which is widely prescribed in oriental medicine for respiratory diseases. This method was validated for linearity, sensitivities (LOD and LOQ), accuracy, and precision. In a practical trial with a BPT sample, hydroxymethylfurfural, a major component of *R. glutinosa*, was detected in the highest abundance. We believe that the knowledge developed in this study will be useful for efficacy studies.

## Figures and Tables

**Table 1:** Parameters for UPLC–MS/MS MRM of the 18 marker compounds.

Analyte ^1^	Ion Mode	Molar Mass (g/mol)	MRM Transition		Cone Voltage (V)	Collision Energy (eV)
			Precursor Ion (Q1) (*m*/*z*)	Production Ion (Q3) (*m*/*z*)		
1	Positive	568.5	569.7	245.0	30	20
2	Positive	126.1	127.0	109.0	20	10
3	Negative	354.3	353.4	191.0	20	20
4	Negative	610.5	609.6	300.5	45	30
5	Positive	446.4	447.5	285.1	30	20
6	Negative	464.4	463.5	300.0	40	20
7	Negative	516.4	515.4	173.0	30	30
8	Negative	516.5	515.0	352.7	30	15
9	Negative	801.0	800.6	636.8	50	20
10	Negative	228.2	227.2	185.0	30	20
11	Negative	284.3	283.4	267.9	30	20
12	Positive	302.2	303.0	153.0	40	30
13	Positive	286.2	286.9	153.2	45	30
14	Negative	1109.3	1107.7	179.0	50	45
15	Positive	785.0	785.3	142.9	15	20
16	Positive	432.5	433.5	415.0	25	13
17	Positive	416.5	417.1	399.0	20	10
18	Positive	400.5	401.1	370.1	35	20

^1^ Mulberroside A (1), hydroxymethylfurfural (2), chlorogenic acid (3), rutin (4), calycosin 7-O-glucoside (5), isoquercetin (6), isochlorogenic acid B (7), isochlorogenic acid A (8), ginsenoside Rg_1_ (9), resveratrol (10), calycosin (11), quercetin (12), kaempferol (13), ginsenoside Rb_1_ (14), astragaloside IV (15), schizandrin (16), gomisin A (17), and gomisin N (18).

**Table 2:** Retention times, linear ranges, regression equations, coefficients of determination (*r*^2^), limits of detection (LOD), and limits of quantitation (LOQ) for the simultaneous determination of the 18 marker compounds using the UPLC–MS/MS MRM method.

Analyte ^1^	Retention Time (min)	Linear Range (μg/L)	Regression Equation ^2^ y=ax+b	*r* ^2^	LOD (μg/L)	LOQ (μg/L)
1	1.03	10.00–500.00	*y* = 485,931.00*x* + 14.47	0.9954	1.05	3.15
2	1.13	100.00–1600.00	*y* = 8870.53*x* + 48,805.00	0.9951	1.10	3.29
3	1.19	10.00–500.00	*y* = 2589.01*x* + 8562.76	0.9984	0.36	1.07
4	1.51	10.00–500.00	*y* = 703.06*x* − 494.34	0.9974	1.97	5.92
5	1.63	100.00–5000.00	*y* = 22,617.20*x* + 152,151.00	0.9992	1.29	3.87
6	1.76	10.00–500.00	*y* = 1375.21*x* − 20.12	0.9974	2.99	8.97
7	1.99	10.00–500.00	*y* = 196.41*x* − 1915.30	0.9959	1.82	5.47
8	2.10	10.00–500.00	*y* = 2282.64*x* − 8840.91	0.9968	0.58	1.73
9	2.75	100.00–2500.00	*y* = 1.28*x* − 45.22	0.9967	24.45	73.35
10	2.96	10.00–500.00	*y* = 819.62*x* − 1368.80	0.9961	0.42	1.26
11	3.44	10.00–500.00	*y* = 12,088.30*x* + 168,161.00	0.9996	0.98	2.95
12	3.46	10.00–500.00	*y* = 2785.91*x* − 22,041.70	0.9981	0.79	2.37
13	4.34	10.00–500.00	*y* = 4723.05*x* + 18,591.70	0.9985	0.40	1.21
14	4.37	10.00–500.00	*y* = 12.96*x* − 150.02	0.9966	2.77	8.31
15	5.16	10.00–500.00	*y* = 25.47*x* − 159.67	0.9959	1.94	5.83
16	6.78	10.00–500.00	*y* = 25,945.60*x* + 306,489.00	0.9995	1.07	3.20
17	7.42	10.00–500.00	*y* = 1279.18*x* + 3833.31	0.9970	2.02	6.07
18	11.28	10.00–500.00	*y* = 5225.76*x* + 79,203.7	0.9993	1.38	4.13

^1^ Mulberroside A (1), hydroxymethylfurfural (2), chlorogenic acid (3), rutin (4), calycosin 7-O-glucoside (5), isoquercetin (6), isochlorogenic acid B (7), isochlorogenic acid A (8), ginsenoside Rg_1_ (9), resveratrol (10), calycosin (11), quercetin (12), kaempferol (13), ginsenoside Rb_1_ (14), astragaloside IV (15), schizandrin (16), gomisin A (17), and gomisin N (18). ^2^ *y*: peak area of compounds; *x*: concentration (μg/L) of compounds.

**Table 3:** Recovery, precision, and stability data of the 18 marker compounds for the UPLC–MS/MS MRM method.

Analyte ^1^	Spiked Amount (μg/L)	Recovery (*n* = 5)		Precision (*n* = 5)				Repeatability (*n* = 6)		Stability
				Intraday		Interday				
		Mean (%)	RSD (%)	Precision (%)	Accuracy (%)	Precision (%)	Accuracy (%)	RSD (%) of RT ^2^	RSD (%) of PA ^3^	RSD (%)
1	80.00	97.74	1.03	1.04	98.43	1.02	97.50	0.22	2.98	1.10
	160.00	98.34	2.02	2.71	96.04	2.44	96.52			
	320.00	97.42	3.62	3.78	94.96	2.95	96.98			
2	200.00	102.02	0.79	3.82	100.56	2.31	100.98	0.27	1.28	0.88
	400.00	100.46	2.85	5.91	102.67	3.86	101.38			
	800.00	103.87	1.75	2.11	104.06	1.80	104.22			
3	20.00	103.15	2.83	3.96	99.43	2.84	101.71	0.24	4.15	1.81
	40.00	98.44	2.54	5.63	100.14	4.03	98.49			
	80.00	96.84	5.04	4.62	96.36	3.94	95.13			
4	20.00	101.50	2.73	2.84	98.69	2.61	100.44	0.30	2.53	1.54
	40.00	95.84	2.20	2.12	97.17	2.72	96.03			
	80.00	90.41	3.50	1.66	98.24	2.57	92.02			
5	200.00	99.40	0.68	2.22	98.51	1.14	99.32	0.16	2.51	0.78
	400.00	104.07	1.88	3.50	102.17	2.95	102.72			
	800.00	108.82	3.24	1.83	100.72	2.41	105.10			
6	20.00	103.48	1.55	2.68	101.68	2.33	102.93	0.21	3.97	1.05
	40.00	92.61	1.88	1.67	95.46	2.29	93.11			
	80.00	86.74	2.07	2.73	93.36	2.76	88.43			
7	40.00	101.02	1.87	1.67	99.98	1.78	100.80	0.42	3.52	0.76
	80.00	98.83	2.14	2.69	97.35	2.63	98.58			
	160.00	96.63	1.99	1.86	94.97	2.55	95.04			
8	40.00	103.15	3.19	2.77	101.59	2.89	101.87	0.24	2.00	1.18
	80.00	92.81	4.16	3.20	96.12	3.21	93.79			
	160.00	88.46	5.34	2.01	92.36	3.54	89.26			
9	400.00	91.82	4.25	4.53	92.46	4.07	92.37	0.39	4.49	0.58
	800.00	85.19	3.16	4.29	104.02	3.44	91.23			
	1600.00	85.23	5.99	5.31	102.78	3.07	87.91			
10	20.00	101.70	6.60	5.82	98.96	5.73	101.73	0.40	2.83	2.91
	40.00	94.60	5.97	1.58	96.38	3.61	95.21			
	80.00	100.83	3.05	2.85	96.30	3.25	97.50			
11	20.00	102.34	2.50	1.31	103.38	2.01	101.92	0.19	2.73	1.42
	40.00	93.98	1.92	3.49	97.79	2.88	94.62			
	80.00	86.64	2.27	1.69	93.40	2.26	88.73			
12	80.00	101.01	1.30	3.06	98.99	2.04	100.58	0.23	4.46	1.47
	160.00	96.98	1.99	3.99	103.94	2.40	99.66			
	320.00	100.12	3.87	3.38	103.27	3.27	100.78			
13	40.00	100.58	0.32	1.09	100.16	0.75	100.62	0.20	2.37	0.46
	80.00	100.38	0.66	1.43	101.29	1.14	100.35			
	160.00	99.59	1.80	1.29	99.57	1.37	99.81			
14	80.00	105.50	1.50	3.44	102.17	1.86	104.33	0.11	5.78	1.88
	160.00	95.64	3.24	3.33	96.85	2.86	94.97			
	320.00	91.82	3.30	3.02	97.82	3.47	92.14			
15	20.00	103.69	1.44	2.22	102.24	1.66	101.61	0.16	5.80	2.67
	40.00	101.57	3.65	4.02	97.88	4.09	99.31			
	80.00	101.07	5.36	4.17	97.15	4.48	99.70			
16	20.00	98.81	0.95	1.87	96.05	1.40	97.68	0.04	1.17	1.28
	40.00	106.71	1.00	1.48	104.08	1.69	105.27			
	80.00	107.58	1.26	1.05	104.94	0.93	105.99			
17	40.00	98.14	1.26	2.19	94.88	1.64	97.43	0.08	1.13	2.29
	80.00	101.65	0.77	3.13	102.41	2.30	101.67			
	160.00	104.13	0.68	0.96	104.55	0.89	103.57			
18	4.00	112.50	3.14	9.77	89.75	4.70	106.25	0.09	2.00	2.97
	8.00	109.25	3.85	9.01	96.93	5.13	106.39			
	16.00	110.25	2.74	2.34	96.25	2.23	106.25			

^1^ Mulberroside A (1), hydroxymethylfurfural (2), chlorogenic acid (3), rutin (4), calycosin 7-O-glucoside (5), isoquercetin (6), isochlorogenic acid B (7), isochlorogenic acid A (8), ginsenoside Rg_1_ (9), resveratrol (10), calycosin (11), quercetin (12), kaempferol (13), ginsenoside Rb_1_ (14), astragaloside IV (15), schizandrin (16), gomisin A (17), and gomisin N (18). ^2^ RT: retention time. ^3^ PA: peak area.

**Table 4:** Amounts of the 18 marker compounds in a BPT sample, determined using the UPLC–MS/MS MRM method.

Analyte ^1^	Mean (mg/g)	SD ^2^ (×10^–2^) (mg/g)	RSD (%)	Source
1	0.71	2.33	3.29	*M. alba*
2	3.20	27.93	8.74	*R. glutinosa*
3	0.36	2.14	5.90	*M. alba* and *A. tataricus*
4	0.01	0.01	1.39	*M. alba*
5	0.08	0.13	1.78	*A. propinquus*
6	0.01	0.01	1.48	*M. alba*
7	0.03	0.03	0.95	*A. tataricus*
8	0.14	0.33	2.31	*A. tataricus*
9	0.07	0.60	8.41	*P. ginseng*
10	0.001	0.01	4.01	*M. alba*
11	0.01	0.02	1.67	*A. propinquus*
12	0.03	0.02	0.84	*A. tataricus*
13	0.03	0.06	1.78	*A. tataricus*
14	0.03	0.22	7.65	*P. ginseng*
15	0.01	0.08	9.17	*A. propinquus*
16	0.11	0.19	1.71	*S. chinensis*
17	0.02	0.07	3.73	*S. chinensis*
18	<LOQ	–	–	*S. chinensis*

^1^ Mulberroside A (1), hydroxymethylfurfural (2), chlorogenic acid (3), rutin (4), calycosin 7-O-glucoside (5), isoquercetin (6), isochlorogenic acid B (7), isochlorogenic acid A (8), ginsenoside Rg_1_ (9), resveratrol (10), calycosin (11), quercetin (12), kaempferol (13), ginsenoside Rb_1_ (14), astragaloside IV (15), schizandrin (16), gomisin A (17), and gomisin N (18). ^2^ SD: standard deviation.

**Table 5:** Operating conditions for the UPLC–MS/MS multiple-reaction monitoring (MRM) simultaneous analysis of BPT.

UPLC Conditions				MS Conditions	
UPLC system	Acquity UPLC I-Class Plus			MS system	Xevo TQ-XS
Column	Acquity UPLC BEH C_18_ column (2.1 mm × 100 mm, particle size: 1.7 μm)			MS software	MassLynx v4.2
Column temperature	45 °C			Ion source ^1^	ESI^+^ or ESI^–^
Sample temperature	5 °C			Acquisition mode	MRM
Injection volume	2.0 μL			Capillary voltage	1.2 kV
Flow rate	0.3 mL/min			Cone gas flow	150 L/h
Mobile phase A	0.1% (*v*/*v*) formic acid in distilled water			Desolvation gas flow	700 L/h
Mobile phase B	Acetonitrile			Desolvation temperature	500 °C
Gradient program of mobile phase	Time (min)	A (%)	B (%)	Source temperature	150 °C
	Initial	80	20		
	14.0	5	95		
	15.0	0	100		
	15.1	80	20		
	18.0	80	20		

^1^ ESI; electrospray ionization.

## Data Availability

All data of this study can be found in this paper.

## Supplementary Materials

The following supporting information can be downloaded at https://www.mdpi.com/article/10.3390/ph17030352/s1: Figure S1: Total ion chromatograms of a blank UPLC–MS/MS MRM trial in positive- and negative-ion modes; Figure S2: Extracted ion chromatograms of each standard compound (A) and of a BPT sample (B) obtained via the UPLC–MS/MS MRM method in positive- or negative-ion modes; Figure S3: Precursor ion (Q1) and product ion (Q3) peaks for each marker compound. Mulberroside A (A), hydroxymethylfurfural (B), chlorogenic acid (C), rutin (D), calycosin 7-O-glucoside (E), isoquercetin (F), 3,4-dicaffeoylquinic acid (G), 3,5-dicaffeoylquinic acid (H), ginsenoside Rg_1_ (I), resveratrol (J), calycosin (K), quercetin (L), kaempferol (M), ginsenoside Rb_1_ (N), astragaloside IV (O), schizandrin (P), gomisin A (Q) and gomisin N (R); Figure S4: Chemical structures of the 18 marker compounds selected for simultaneous determination in BPT; Table S1: Information about the 18 reference standard compounds.
