# Supplementary material for: An Ultra-Performance Liquid Chromatography–Tandem Mass Spectrometric Method for the Simultaneous Determination of Eighteen Marker Compounds in the Traditional Herbal Formula Bopyeo-Tang
Source: Pharmaceuticals (Basel). 2024 Mar 8;17(3):352. doi: 10.3390/ph17030352 (PMC10974790; doi:10.3390/ph17030352)
Supplement: Supplementary file 1 [file pharmaceuticals-17-00352-s001.zip › pharmaceuticals-Supplementary Materials.pdf]

Table S1

Information about the 18 reference standard compounds.

Analyte[1]	Purity (%)	Molecular formula	CAS No.	PubChem CID	Catalog No.	Maker
1	98.1	$C_{26}H_{32}O_{14}$	102841-42-9	6443484	ES030-A	EnsolBioSciences
2	≥99.0	$C_6H_6O_3$	67-47-0	237332	W501808	Merck KGaA
3	99.7	$C_{16}H_{18}O_9$	327-97-9	1794427	PHL89175	Merck KGaA
4	97.2	$C_{27}H_{30}O_{16}$	153-18-4	5280805	89270	Merck KGaA
5	99.4	$C_{22}H_{22}O_{10}$	20633-67-4	5318267	DR10682	Shanghai Sunny Biotech
6	99.2	$C_{21}H_{20}O_{12}$	482-35-9	5280804	BP0973	Biopurify Phytochemicals
7	98.3	$C_{25}H_{24}O_{12}$	14534-61-3	5281780	DR11569	Shanghai Sunny Biotech
8	98.2	$C_{25}H_{24}O_{12}$	2450-53-5	6474310	DR11570	Shanghai Sunny Biotech
9	99.8	$C_{42}H_{72}O_{14}$	22427-39-0	441923	DR10697	Shanghai Sunny Biotech
10	100.0	$C_{14}H_{12}O_3$	501-36-0	445154	R5010	Merck KGaA
11	99.1	$C_{16}H_{12}O_5$	20575-57-9	5280448	DR10681	Shanghai Sunny Biotech
12	99.2	$C_{15}H_{10}O_7$	117-39-5	5280343	CFN99272	Wuhan ChemFaces Biochemical
13	≥98.0	$C_{15}H_{10}O_6$	520-18-3	5280863	DR10770	Shanghai Sunny Biotech
14	98.5	$C_{54}H_{92}O_{23}$	41753-43-9	9898279	DR10691	Shanghai Sunny Biotech
15	98.6	$C_{41}H_{68}O_{14}$	84687-43-4	13943297	BP0213	Biopurify Phytochemicals
16	99.3	$C_{24}H_{32}O_7$	7432-28-2	23915	BP1265	Biopurify Phytochemicals
17	99.9	$C_{23}H_{28}O_7$	58546-54-6	634470	DR100561	Shanghai Sunny Biotech
18	99.2	$C_{23}H_{28}O_6$	69176-52-9	158103	DR10557	Shanghai Sunny Biotech

[1] Mulberroside A (1), hydroxymethylfurfural (2), chlorogenic acid (3), rutin (4), calycosin 7-O-glucoside (5), isoquercetin (6), 3,4-dicaffeoylquinic acid (7), 3,5-dicaffeoylquinic acid (8), ginsenoside Rg_1 (9), resveratrol (10), calycosin (11), quercetin (12), kaempferol (13), ginsenoside Rb_1 (14), astragaloside IV (15), schizandrin (16), gomisin A (17) and gomisin N (18).

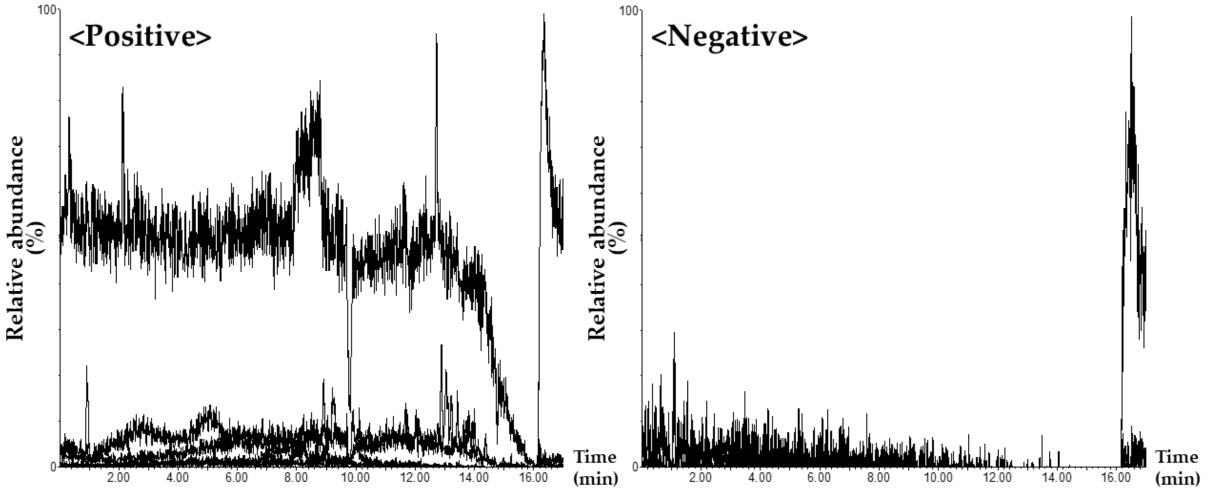

Figure S1. Total ion chromatograms of a blank UPLC–MS/MS MRM trial in positive and negative ion modes.

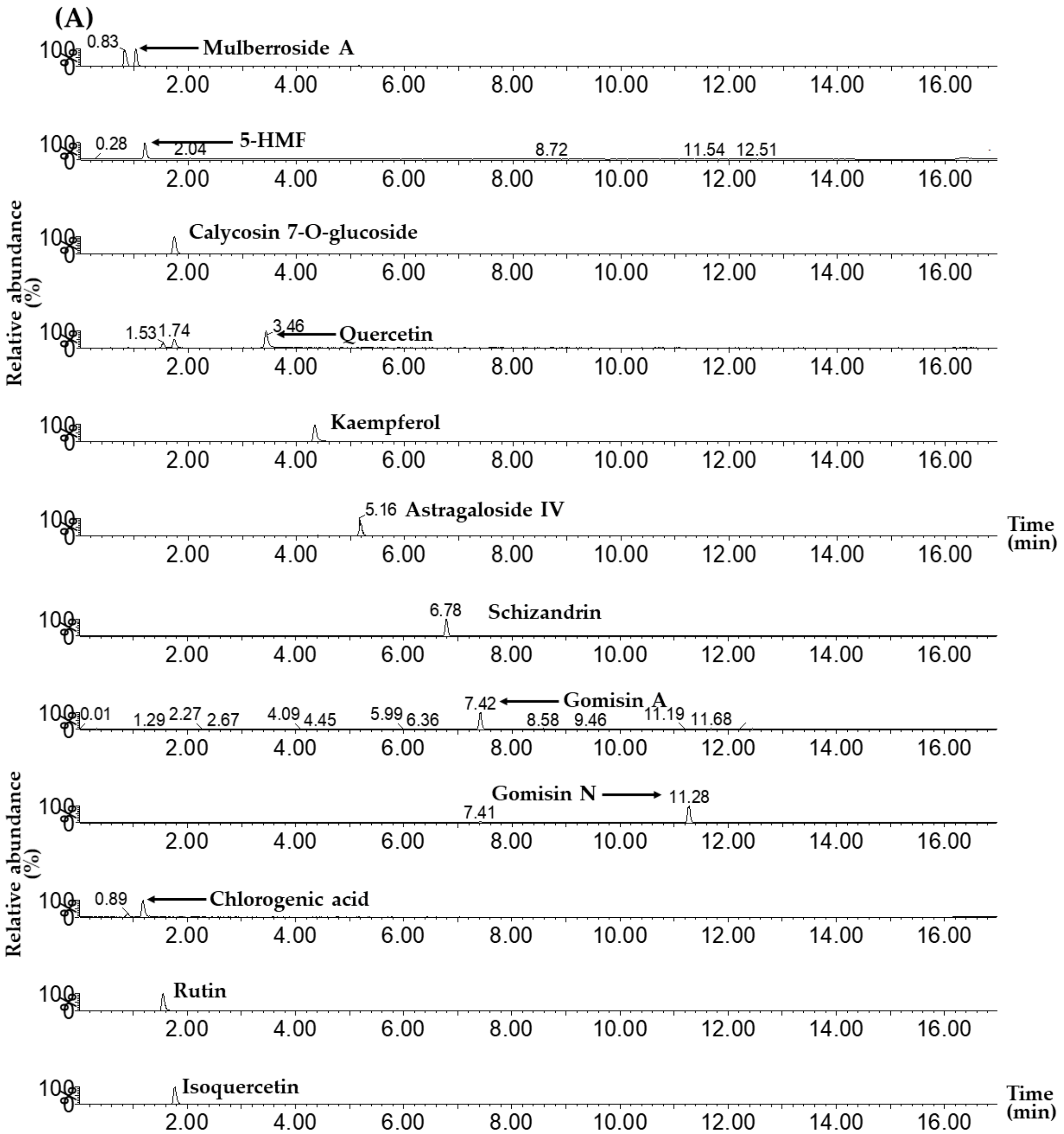
(A)
Mulberroside A
5-HMF
Calycosin 7-O-glucoside
Quercetin
Kaempferol
Astragaloside IV
Schizandrin
Gomisin A
Gomisin N
Chlorogenic acid
Rutin
Isoquercetin
Relative abundance (%)
Time (min)

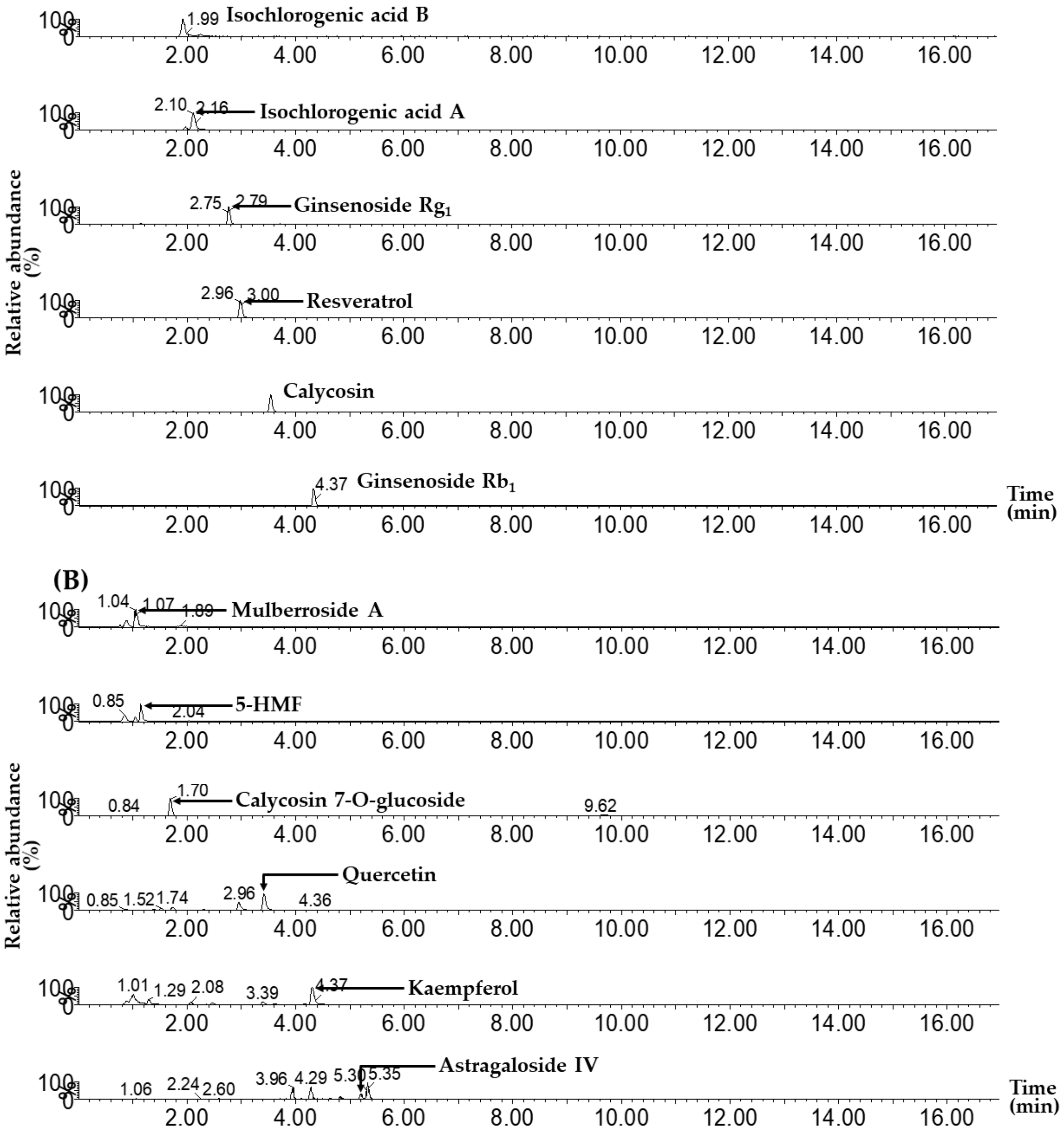
Isochlorogenic acid B
1.99
Isochlorogenic acid A
2.10
2.16
Ginsenoside Rg1
2.75
2.79
Resveratrol
2.96
3.00
Calycosin
Ginsenoside Rb1
4.37
Relative abundance (%)
100
0
2.00
4.00
6.00
8.00
10.00
12.00
14.00
16.00
Time (min)
(B)
Mulberroside A
1.04
1.07
1.89
5-HMF
0.85
2.04
Calycosin 7-O-glucoside
0.84
1.70
9.62
Quercetin
0.85
1.52
1.74
2.96
4.36
Kaempferol
1.01
1.29
2.08
3.39
4.37
Astragaloside IV
1.06
2.24
2.60
3.96
4.29
5.30
5.35

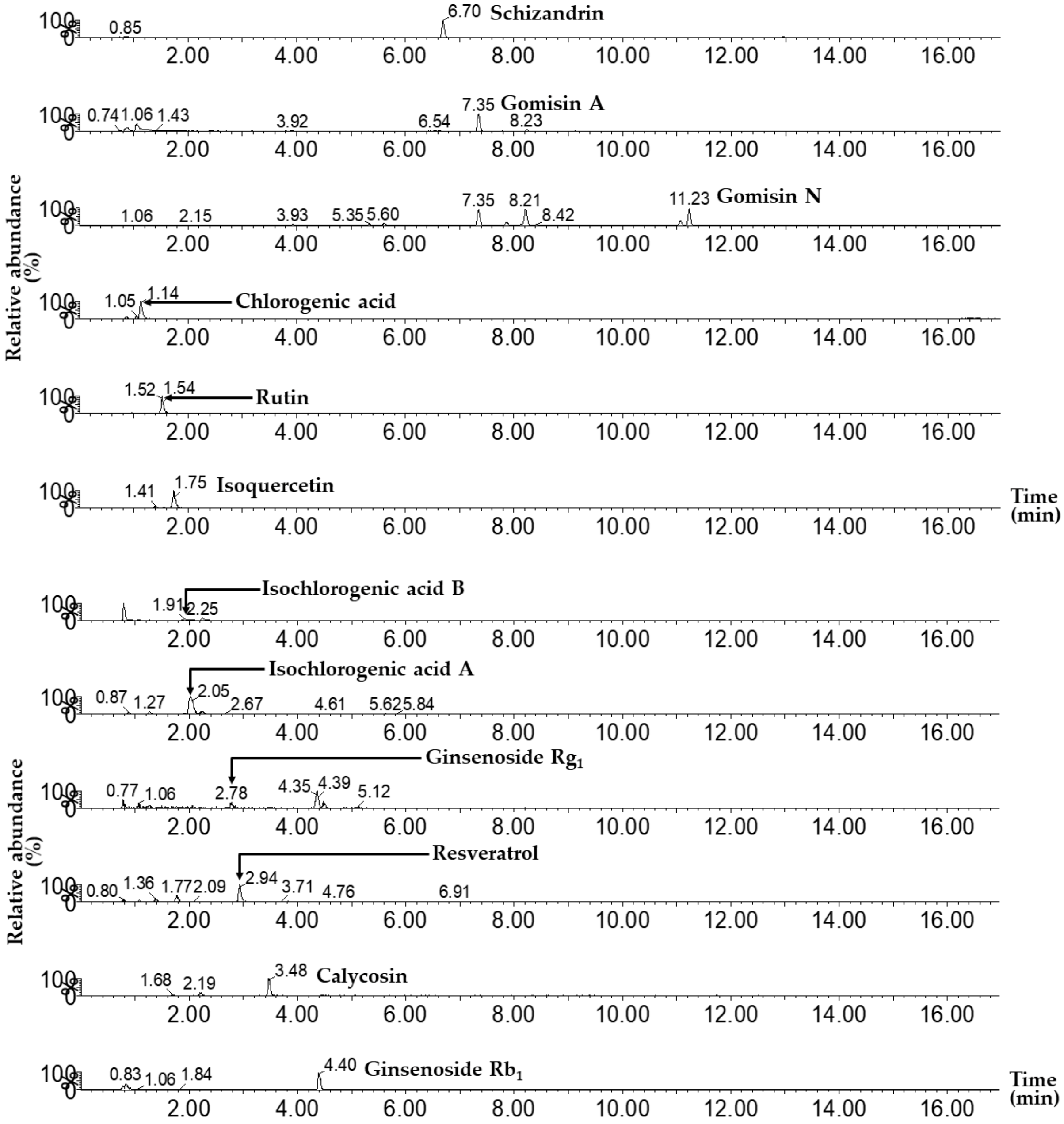

Figure S2. Extracted ion chromatograms of each standard compound (A) and of a BPT sample (B) by the UPLC–MS/MS MRM method in positive or negative ion modes.

(A)

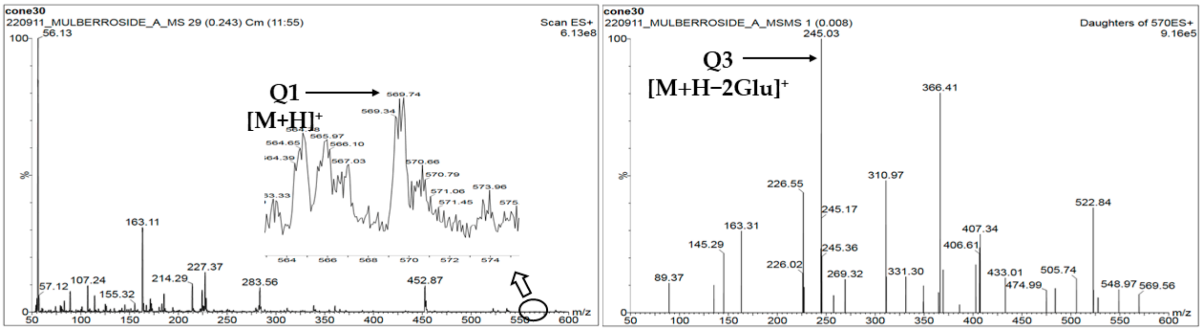
cone30
220911_MULBERROSIDE_A_MS 29 (0.243) Cm (11:55)
Scan ES+
6.13e8
Q1
[M+H]+
cone30
220911_MULBERROSIDE_A_MSMS 1 (0.008)
Daughters of 570ES+
9.16e5
Q3
[M+H−2Glu]+
m/z

(B)

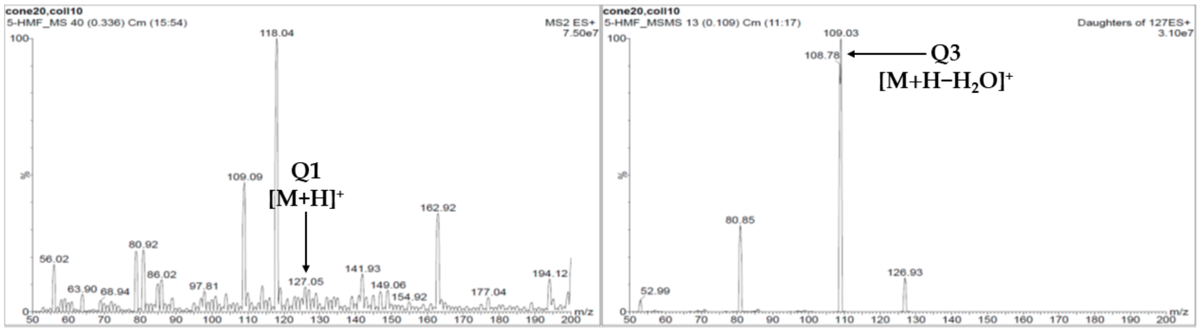
cone20,coll10
5-HMF_MS 40 (0.336) Cm (15:54)
MS2 ES+
7.50e7
Q1
[M+H]+
cone20,coll10
5-HMF_MSMS 13 (0.109) Cm (11:17)
Daughters of 127ES+
3.10e7
Q3
[M+H−H2O]+
m/z

(C)

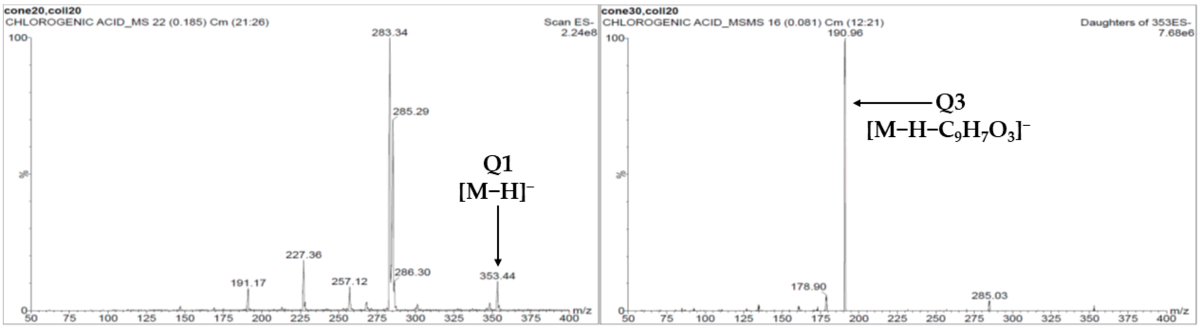
cone20,coll20
CHLOROGENIC ACID_MS 22 (0.185) Cm (21:26)
Scan ES-
2.24e8
Q1
[M−H]−
cone30,coll20
CHLOROGENIC ACID_MSMS 16 (0.081) Cm (12:21)
Daughters of 353ES-
7.68e6
Q3
[M−H−C9H7O3]−
m/z

(D)

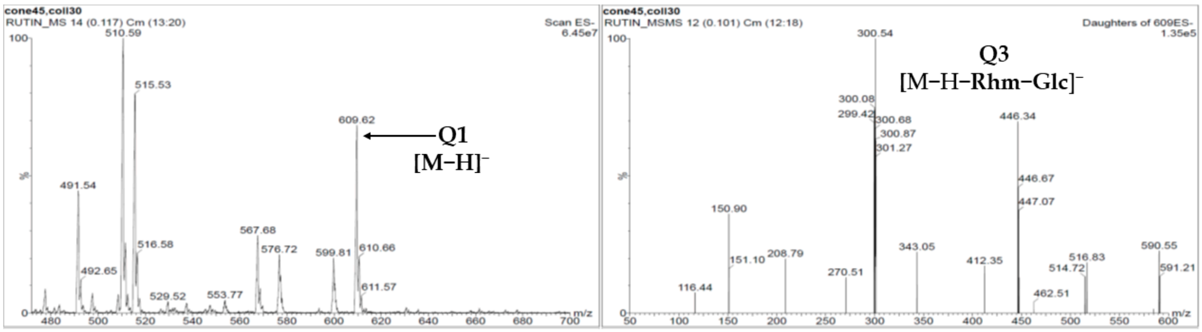
cone45,coll30
RUTIN_MS 14 (0.117) Cm (13:20)
Scan ES-
6.45e7
Q1
[M−H]−
cone45,coll30
RUTIN_MSMS 12 (0.101) Cm (12:18)
Daughters of 609ES-
1.35e5
Q3
[M−H−Rhm−Glc]−
m/z

(E)

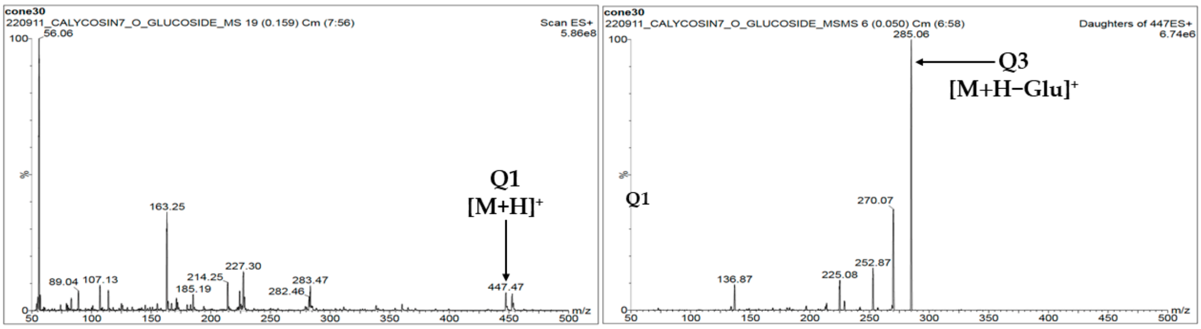
cone30
220911_CALYCOSIN7_O_GLUCOSIDE_MS 19 (0.159) Cm (7:56)
Scan ES+
5.86e8
56.06
163.25
89.04
107.13
185.19
214.25
227.30
282.46
283.47
Q1
[M+H]+
447.47
m/z
cone30
220911_CALYCOSIN7_O_GLUCOSIDE_MSMS 6 (0.050) Cm (6:58)
Daughters of 447ES+
6.74e6
285.06
Q3
[M+H−Glu]+
Q1
270.07
136.87
225.08
252.87
m/z

(F)

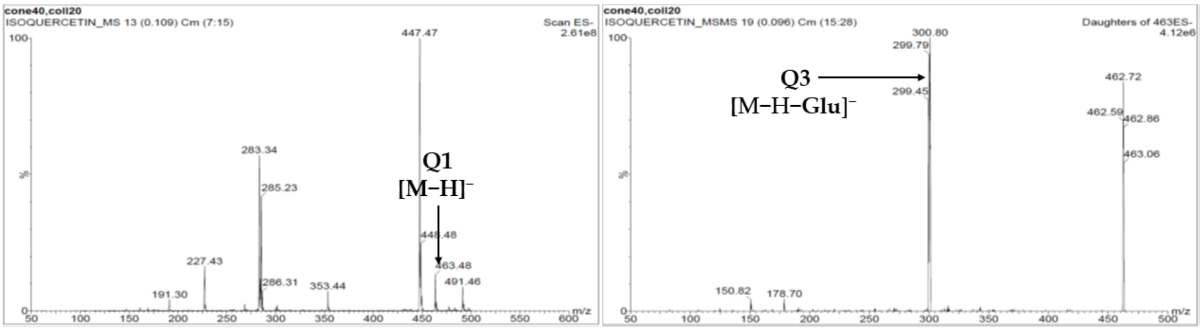
cone40,coll20
ISOQUERCETIN_MS 13 (0.109) Cm (7:15)
Scan ES-
2.61e8
447.47
283.34
285.23
Q1
[M−H]−
227.43
191.30
286.31
353.44
463.48
491.46
m/z
cone40,coll20
ISOQUERCETIN_MSMS 19 (0.096) Cm (15:28)
Daughters of 463ES-
4.12e6
300.80
299.79
Q3
[M−H−Glu]−
299.45
462.72
462.59
462.86
463.06
150.82
178.70
m/z

(G)

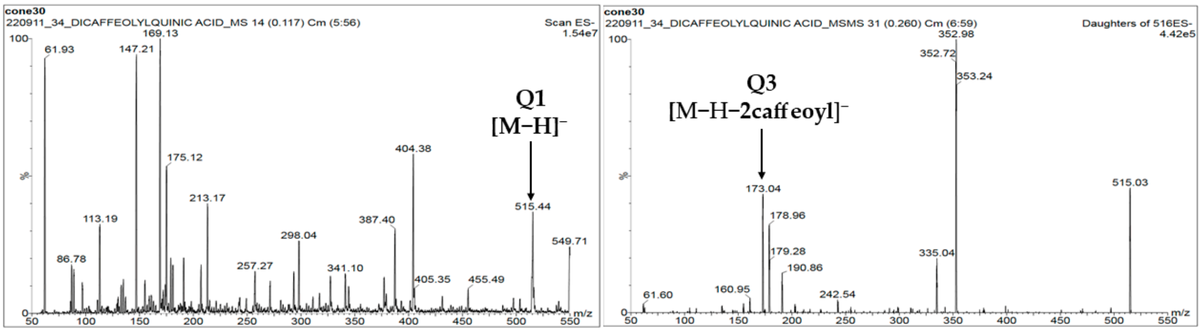
cone30
220911_34_DICAFFEOLYLQUINIC ACID_MS 14 (0.117) Cm (5:56)
Scan ES-
1.54e7
169.13
61.93
147.21
Q1
[M−H]−
175.12
404.38
213.17
515.44
113.19
387.40
298.04
549.71
86.78
257.27
341.10
405.35
455.49
m/z
cone30
220911_34_DICAFFEOLYLQUINIC ACID_MSMS 31 (0.260) Cm (6:59)
Daughters of 516ES-
4.42e5
352.98
352.72
353.24
Q3
[M−H−2caffeoyl]−
173.04
515.03
178.96
179.28
190.86
335.04
61.60
160.95
242.54
m/z

(H)

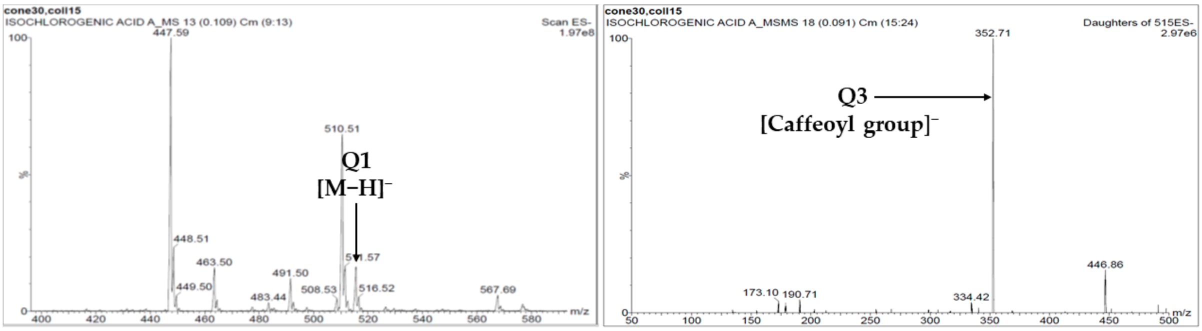
cone30,coll15
ISOCHLOROGENIC ACID A_MS 13 (0.109) Cm (9:13)
Scan ES-
1.97e8
447.59
510.51
Q1
[M−H]−
448.51
463.50
449.50
491.50
508.53
516.52
483.44
567.69
m/z
cone30,coll15
ISOCHLOROGENIC ACID A_MSMS 18 (0.091) Cm (15:24)
Daughters of 515ES-
2.97e6
352.71
Q3
[Caffeoyl group]−
446.86
173.10
190.71
334.42
m/z

(I)

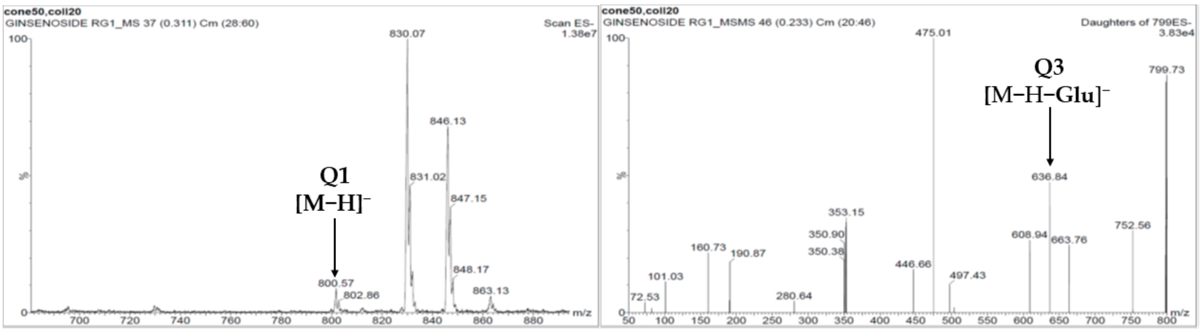
cone50,coll20
GINSENOSIDE RG1_MS 37 (0.311) Cm (28:60)
Scan ES-
1.38e7
Q1
[M−H]⁻
830.07
846.13
831.02
847.15
848.17
863.13
800.57
802.86
cone50,coll20
GINSENOSIDE RG1_MSMS 46 (0.233) Cm (20:46)
Daughters of 799ES-
3.83e4
475.01
Q3
[M−H−Glu]⁻
799.73
636.84
353.15
350.90
350.38
160.73
190.87
101.03
72.53
280.64
446.66
497.43
608.94
663.76
752.56
m/z

(J)

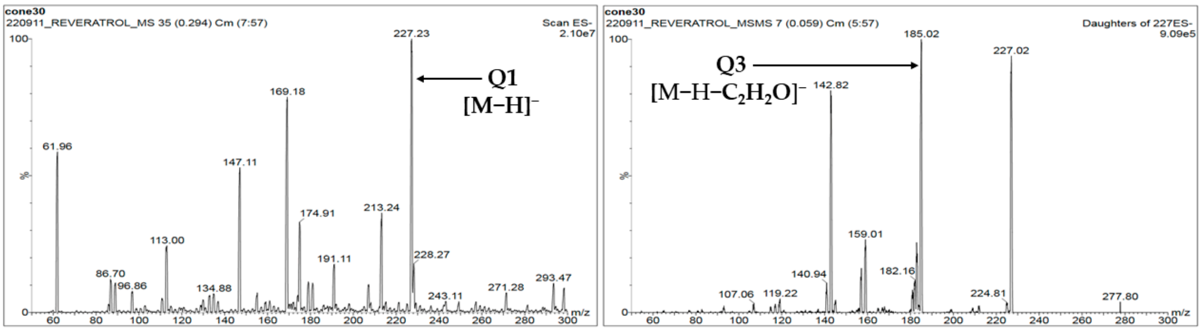
cone30
220911_REVERATROL_MS 35 (0.294) Cm (7:57)
Scan ES-
2.10e7
227.23
Q1
[M−H]⁻
169.18
61.96
147.11
174.91
213.24
113.00
86.70
96.86
134.88
191.11
228.27
243.11
271.28
293.47
cone30
220911_REVERATROL_MSMS 7 (0.059) Cm (5:57)
Daughters of 227ES-
9.09e5
Q3
[M−H−C2H2O]⁻
185.02
227.02
142.82
159.01
140.94
182.16
107.06
119.22
224.81
277.80
m/z

(K)

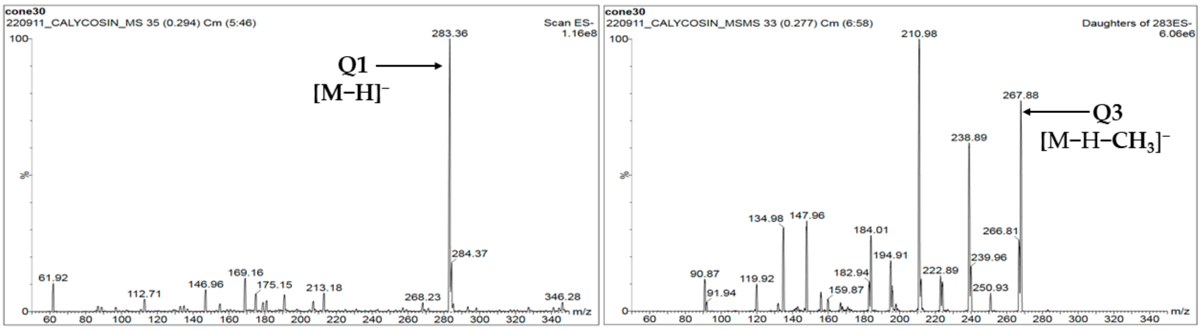
cone30
220911_CALYCOSIN_MS 35 (0.294) Cm (5:46)
Scan ES-
1.16e8
283.36
Q1
[M−H]⁻
284.37
61.92
112.71
146.96
169.16
175.15
213.18
268.23
346.28
cone30
220911_CALYCOSIN_MSMS 33 (0.277) Cm (6:58)
Daughters of 283ES-
6.06e6
210.98
267.88
Q3
[M−H−CH3]⁻
238.89
134.98
147.96
184.01
266.81
194.91
239.96
90.87
91.94
119.92
182.94
159.87
222.89
250.93
m/z

(L)

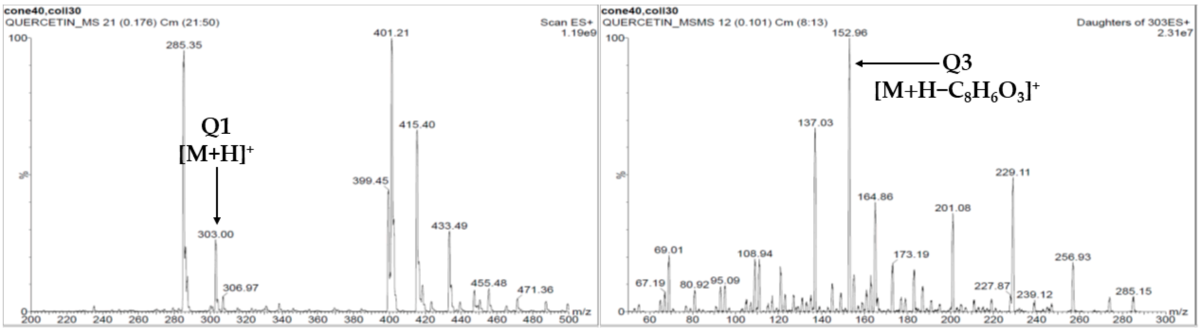
cone40,coll30
QUERCETIN_MS 21 (0.176) Cm (21:50)
Scan ES+
1.19e9
285.35
401.21
415.40
Q1
[M+H]⁺
399.45
303.00
433.49
306.97
455.48
471.36
cone40,coll30
QUERCETIN_MSMS 12 (0.101) Cm (8:13)
Daughters of 303ES+
2.31e7
152.96
Q3
[M+H−C8H6O3]⁺
137.03
229.11
164.86
201.08
69.01
108.94
173.19
256.93
67.19
80.92
95.09
227.87
239.12
285.15
m/z

(M)

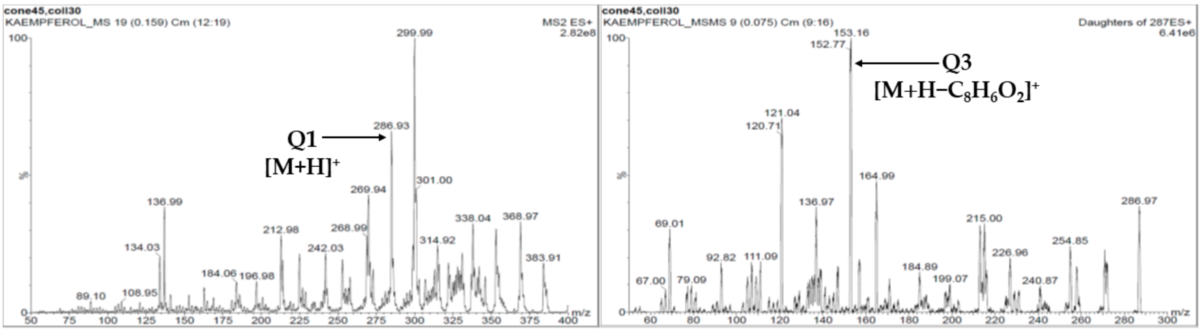
cone45,coll30
KAEMPFEROL_MS 19 (0.159) Cm (12:19)
MS2 ES+
2.82e8
Q1
[M+H]+
cone45,coll30
KAEMPFEROL_MSMS 9 (0.075) Cm (9:16)
Daughters of 287ES+
6.41e6
Q3
[M+H−C8H6O2]+

(N)

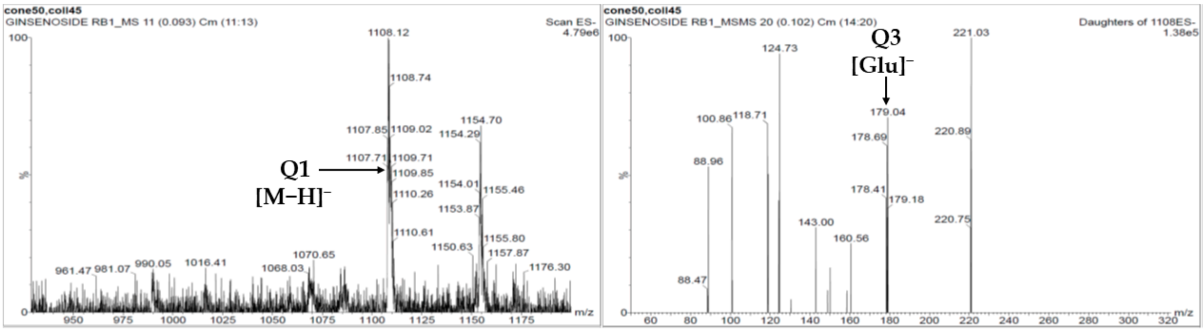
cone50,coll45
GINSENOSIDE RB1_MS 11 (0.093) Cm (11:13)
Scan ES-
4.79e6
Q1
[M−H]−
cone50,coll45
GINSENOSIDE RB1_MSMS 20 (0.102) Cm (14:20)
Daughters of 1108ES-
1.38e5
Q3
[Glu]−

(O)

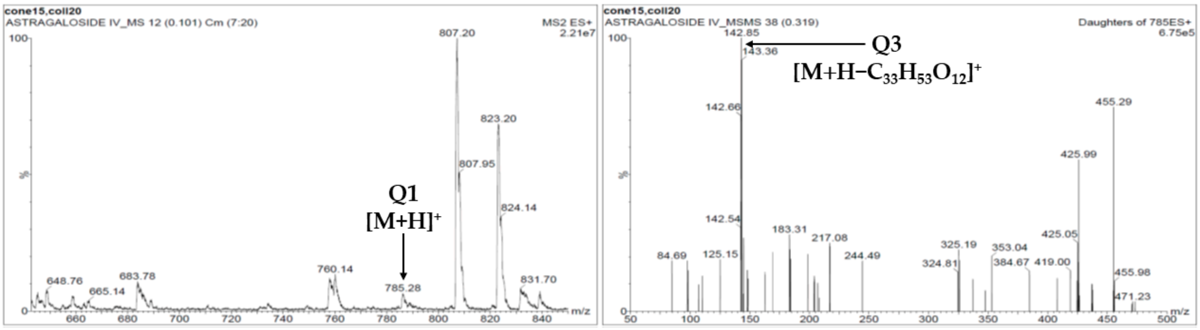
cone15,coll20
ASTRAGALOSIDE IV_MS 12 (0.101) Cm (7:20)
MS2 ES+
2.21e7
Q1
[M+H]+
cone15,coll20
ASTRAGALOSIDE IV_MSMS 38 (0.319)
Daughters of 785ES+
6.75e5
Q3
[M+H−C33H53O12]+

(P)

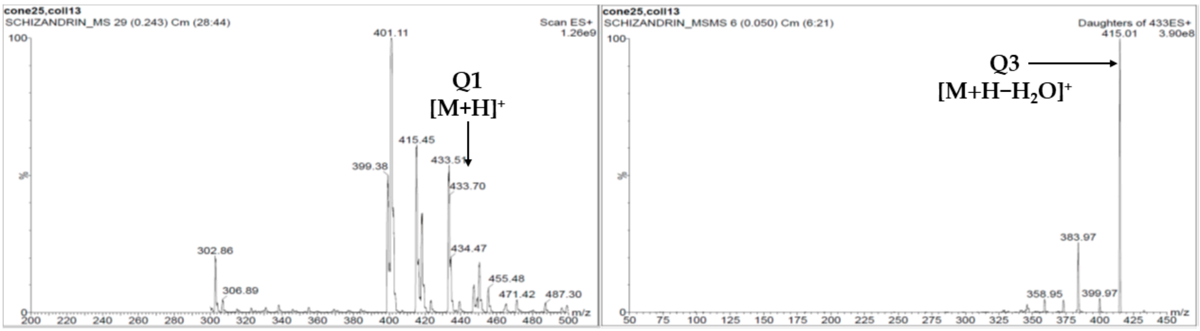
cone25,coll13
SCHIZANDRIN_MS 29 (0.243) Cm (28:44)
Scan ES+
1.26e9
Q1
[M+H]+
cone25,coll13
SCHIZANDRIN_MSMS 6 (0.050) Cm (6:21)
Daughters of 433ES+
3.90e8
Q3
[M+H−H2O]+

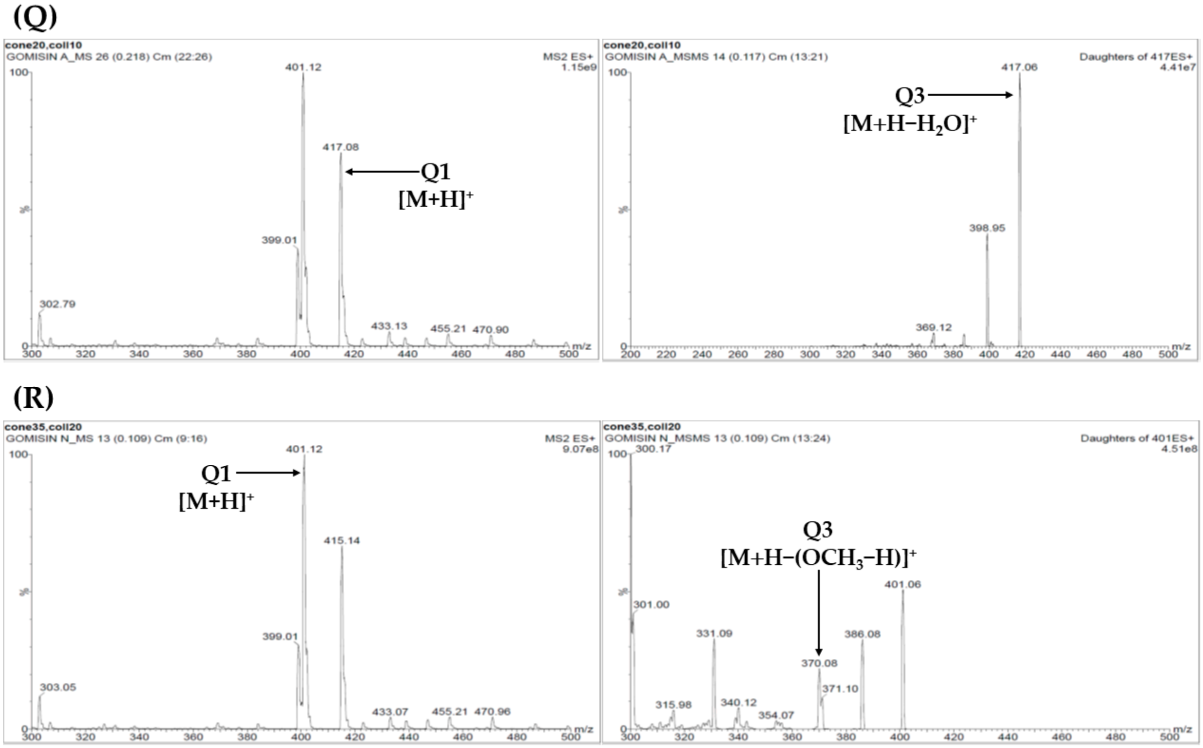

Figure S3. Precursor ion (Q1) and product ion (Q3) peaks for each marker compound. Mulberroside A (A), hydroxymethylfurfural (B), chlorogenic acid (C), rutin (D), calycosin 7-O-glucoside (E), isoquercetin (F), 3,4-dicaffeoylquinic acid (G), 3,5-dicaffeoylquinic acid (H), ginsenoside Rg_1 (I), resveratrol (J), calycosin (K), quercetin (L), kaempferol (M), ginsenoside Rb_1 (N), astragaloside IV (O), schizandrin (P), gomisin A (Q) and gomisin N (R).

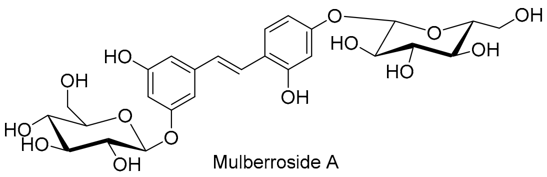

Mulberroside A

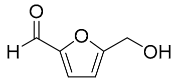

Hydroxymethylfurfural

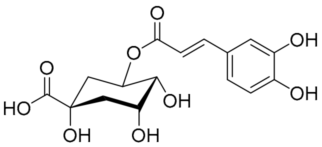

Chlorogenic acid

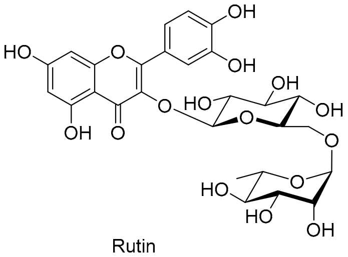

Rutin

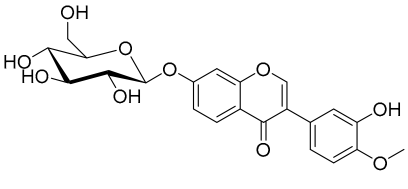

Calycosin 7-O-glucoside

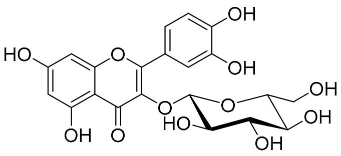

Isoquercetin

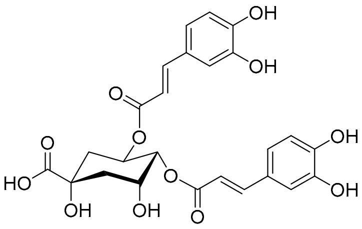

Isochlorogenic acid B

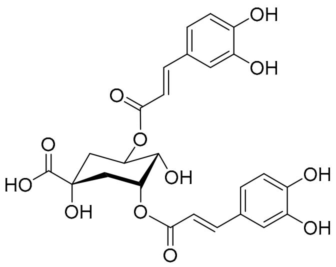

Isochlorogenic acid A

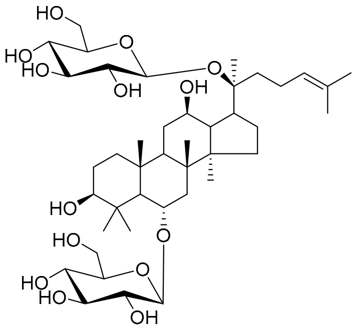

Ginsenoside Rg_1

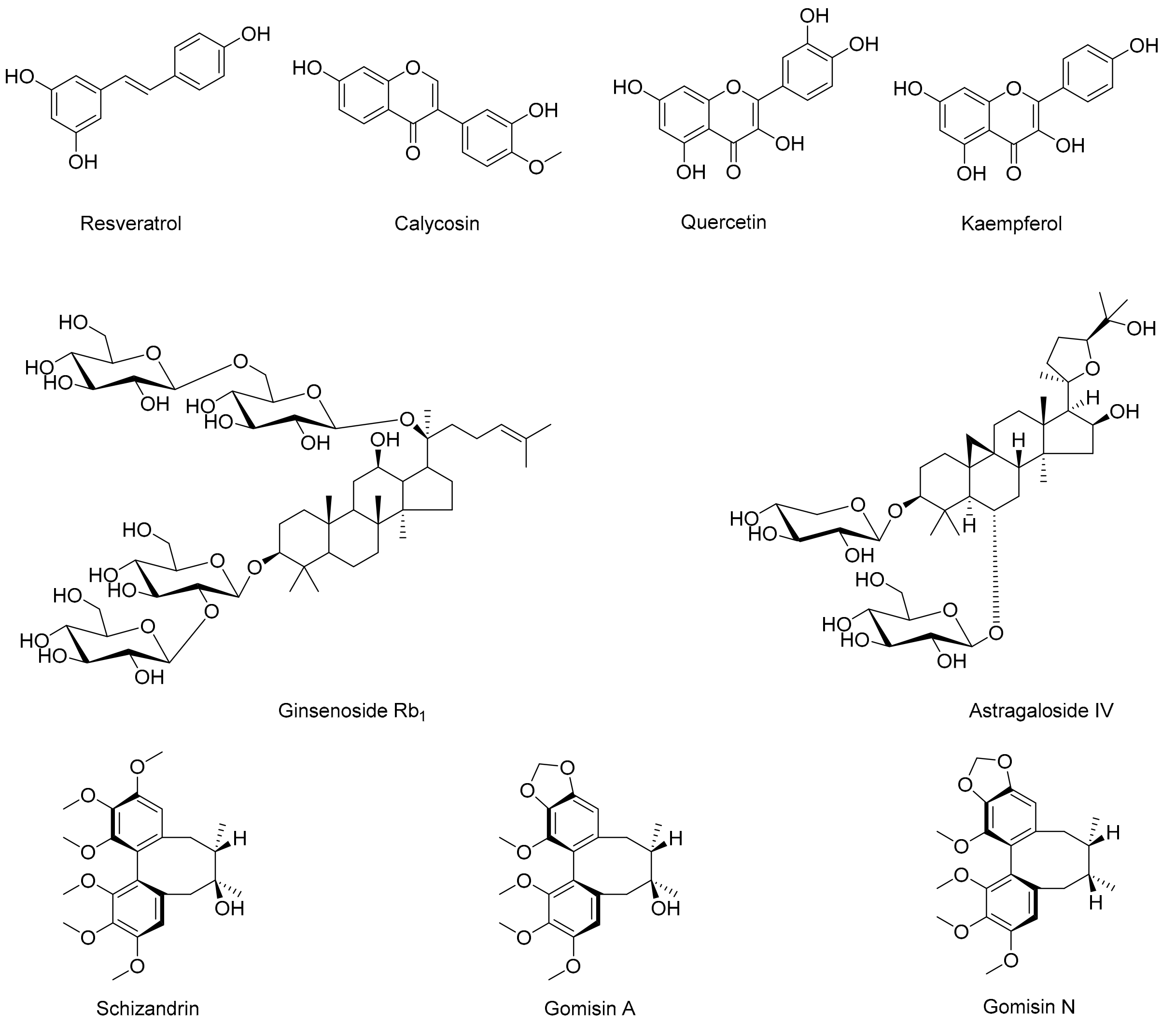

Figure S4. Chemical structures of the 18 marker compounds selected for simultaneous determination in BPT.
